# Classification of elderly pain severity from automated video clip facial action unit analysis: A study from a Thai data repository

**DOI:** 10.3389/frai.2022.942248

**Published:** 2022-10-06

**Authors:** Patama Gomutbutra, Adisak Kittisares, Atigorn Sanguansri, Noppon Choosri, Passakorn Sawaddiruk, Puriwat Fakfum, Peerasak Lerttrakarnnon, Sompob Saralamba

**Affiliations:** ^1^Aging and Aging Palliative Care Research Cluster, Department of Family Medicine, Faculty of Medicine, Chiang Mai University, Chiang Mai, Thailand; ^2^Northern Neuroscience Center, Faculty of Medicine, Chiang Mai University, Chiang Mai, Thailand; ^3^College of Arts, Media, and Technology, Chiang Mai University, Chiang Mai, Thailand; ^4^Department of Anesthesiology, Faculty of Medicine, Chiang Mai University, Chiang Mai, Thailand; ^5^Mahidol-Oxford Tropical Medicine Research Unit, Faculty of Tropical Medicine, Mahidol University, Bangkok, Thailand

**Keywords:** facial action coding system, chronic pain, elderly, dementia, Asian

## Abstract

Data from 255 Thais with chronic pain were collected at Chiang Mai Medical School Hospital. After the patients self-rated their level of pain, a smartphone camera was used to capture faces for 10 s at a one-meter distance. For those unable to self-rate, a video recording was taken immediately after the move that causes the pain. The trained assistant rated each video clip for the pain assessment in advanced dementia (PAINAD). The pain was classified into three levels: mild, moderate, and severe. OpenFace^©^ was used to convert the video clips into 18 facial action units (FAUs). Five classification models were used, including logistic regression, multilayer perception, naïve Bayes, decision tree, k-nearest neighbors (KNN), and support vector machine (SVM). Out of the models that only used FAU described in the literature (FAU 4, 6, 7, 9, 10, 25, 26, 27, and 45), multilayer perception is the most accurate, at 50%. The SVM model using FAU 1, 2, 4, 7, 9, 10, 12, 20, 25, and 45, and gender had the best accuracy of 58% among the machine learning selection features. Our open-source experiment for automatically analyzing video clips for FAUs is not robust for classifying pain in the elderly. The consensus method to transform facial recognition algorithm values comparable to the human ratings, and international good practice for reciprocal sharing of data may improve the accuracy and feasibility of the machine learning's facial pain rater.

## Introduction

### Overview

Pain severity data are crucial for pain management decision-making. However, the accuracy of the assessment of pain in older patients is still challenging. Although self-reported pain ratings are the golden standard, elderly patients have limited cognitive and physical functions, making assessing their pain difficult. In addition, the COVID-19 pandemic and caregiver shortage have made hospital visits challenging. Therefore, home-based pain management becomes essential for these patients. Machine learning integration that can provide automated and continuous pain monitoring at home might be the ideal solution.

Despite their availability, complicated objective pain measurements, such as MRI and heart rate variability, are not feasible and present ethical challenges in the actual clinical setting. Although the facial expression is a visible, informative feature associated with pain severity, several limitations inhibit its real-life application. Furthermore, in the typical clinical setting, patients with chronic pain typically experience persistent and spontaneous pain without physical pain stimuli. In a laboratory setting, getting the appropriate lighting and facial angle is challenging. These factors may impact the performance of the model. The challenge of developing an efficient model necessitates the use of databases that contain samples from different environments where pain may occur and are encrypted according to standards that enable sharing among international collaborations (Prkachin and Hammal, 2021).

### Background

The original facial activity measurement system, the facial action coding system (FACS) (Facial Action Coding System, 2020), requires manual coding, making it time-consuming and costly. Efforts have been made to develop an automated facial analysis algorithm to overcome this limitation. Numerous studies have been conducted to define pain-related facial action units (FAUs) using automated computer vision. Prkachin et al. conducted a benchmark study on pain-related FAUs with the help of picture frames from 129 people experiencing shoulder pain. They rated facial pain during illicit acute pain by motion (Prkachin and Solomon, 2008). They adopted Ekman's coding and used four certified human coders who classified each picture frame into 1–5 levels based on the pain intensity (1 = trace to 5 = maximum). They listed six action units (AUs) that were significantly associated with pain, including brow lowering (AU4), orbital tightening (AU6 and AU7), levator contraction (AU9 and AU10), and eye closure (AU43). A prediction model was proposed using the sum of AU4, AU6, or AU7, whichever is higher, AU9 or AU10, whichever is higher, and AU43 for each AU (Pai*n* = AU4 + AU6 or AU7 + AU9 or AU10 + AU43) (Lucey et al., 2009). A recent systematic review summarized the following AUs as consistently reported as having a connection to pain: AU4, 6, 7, 9, 10, 25, 26, 27, and 43 (Kunz et al., 2019).

OpenFace^©^, a well-known open-source algorithm (Lötsch and Ultsch, 2018), was trained using a dataset dominated by young, healthy Caucasian persons. Patients with shoulder pain were videotaped in a laboratory setting as they experienced illicit pain, according to the widely used UNBC McMaster pain dataset (Prkachin and Solomon, 2008). However, aging-related wrinkles (Kunz et al., 2017), cognitive impairment (Taati et al., 2019), and gender or ethnic-related skin fairness (Buolamwini and Gebru, 2018) might cause the model's representational bias. Furthermore, OpenFace^©^ cannot distinguish AU24–27, which uses the same muscle as AU23 and AU43 (eye closure), and AU45. For each AU, the OpenFace^©^ generates values representing the algorithm's level of confidence that the AU is present. The per-frame label representing an integer value from zero to five is estimated from either classification or regression and the data from computer vision detect FAUs as a time series of continuous values. For each AU, the earlier studies used two methods to estimate points from the time series. The first method used mean measurements for different AUs (Lucey et al., 2009), whereas the second method used time series to determine the area under the FAU pulse curve (Haines et al., 2019). Compared with human FACS coding, the accuracy of the OpenFace^©^ is 90% for constrained images and 80% for real-world images. However, the validity of OpenFace^©^ for detecting pain in the faces of elderly and dementia patients is still debatable. According to one study that used manual code FACs, OpenFace^©^ has a precision of only 54% for AU4 and 70.4% for AU43 (Taati et al., 2019). Meanwhile, the Delaware database project uses OpenFace^©^ to analyze individuals under 30 with a higher proportion of non-Caucasian ethnicity and discovered that it has a precision of 98% for AU4 and 73% for AU45 (Mende-Siedlecki et al., 2020).

Recent studies have attempted to apply other algorithms to clinical pain management. For example, Algamadhi et al. (Alghamdi and Alaghband, 2022) developed a facial expression-based automatic pain assessment system (FEAPAS) to notify the medical staff when a patient is experiencing severe pain and to record the incident and pain level. The convolutional neural network (CNN) algorithm was optimized using the UNBC McMaster pain dataset and demonstrated an accuracy of 99 and 90.5% for the trained and test (unseen) datasets, respectively. However, the author also mentioned the different datasets to confirm the algorithm's performance. According to Lautenbacher et al., the currently available automated facial recognition algorithms, that is, Facereader7, OpenFace^©^, and Affdex SDK, have comparable outcomes with a lack of robustness (0.3–0.4%) and inconsistency between manual and automatic AU detection. In addition, the discrepancy between laboratory-based eliciting of responses and automatic AU coding significantly increases when the facial expression occurs during spontaneous (emotional) eliciting (Lautenbacher et al., 2022).

### Purpose

Despite a rapid increase in literature on automated facial pain recognition, most of the studies have been conducted in western countries, which may imply that the Asian population is underrepresented. Although Asian ethnic people are represented in some studies' datasets, this approach only addresses the different physical prototypes and ignores cultural and institutional aspects of healthcare. According to the communal coping model (CCM) of catastrophizing theoretical framework, personal perception of pain may influence the degree of pain expression to communicate information to others (Tsui et al., 2012). Stereotypically, people in Asian countries are generally reserved in their expression of pain owing to their religious beliefs and the uniformity of their societies (Chen et al., 2008). The current algorithm's reliability in classifying pain in people from Asian countries at a level comparable to that of people from western countries is still debatable. Therefore, our study aims to evaluate the model's accuracy using data from OpenFace^©^ in classifying the level of pain in Asian elderly patients who are receiving chronic pain treatment in an Asian country.

## Methods

### Study design

This is a prospective registry-building and facial expression study on elderly Thai people with chronic pain. The Chiang Mai University Institutional Review Board (CMU IRB no 05429) approved the research, and patients or the designated caregivers provided consent for participation. The G^*^ Power (Faul et al., 2007) determined that a sample size of at least 246 samples is necessary to estimate the proportion of severe pain in the target population with 95% confidence, an error margin of 5%, and an unlimited population size. The assumed severe pain proportion was 0.2, which was based on a previous study of the same population (Gomutbutra et al., 2020).

### Study population

Cases were collected from the pain clinic, internal medicine ward, orthopedic ward, and nursing home institute of Chiang Mai University Hospital between May 2018 and December 2019. In Thailand, patients older than 55 years are eligible for retirement healthcare benefits; therefore, this age is appropriate for recruitment purposes regarding logistic feasibility. Other criteria included a chronic history (more than 3 months) and ongoing pain during the assessment. The participants in this study were patients or caregivers who could communicate in Thai. The clinic's screening nurse asked patients and/or caregivers who either had visited for the first time or had returned for follow-up care if they would be interested in participating in the study. The clinics allowed our research assistant to discuss the study with prospective participants, explain its procedures, express confidence in the video clip data, and request for written consent. Approximately 20% of the invited patients declined to owe to lack time or frailty. Because all participants were volunteers, potential coercion was avoided. The volunteer spent <30 min completing the questionnaire and recording a 10 s video for which they were paid ~7 USD (200 Baths).

### Data collection

**Figure 1:**
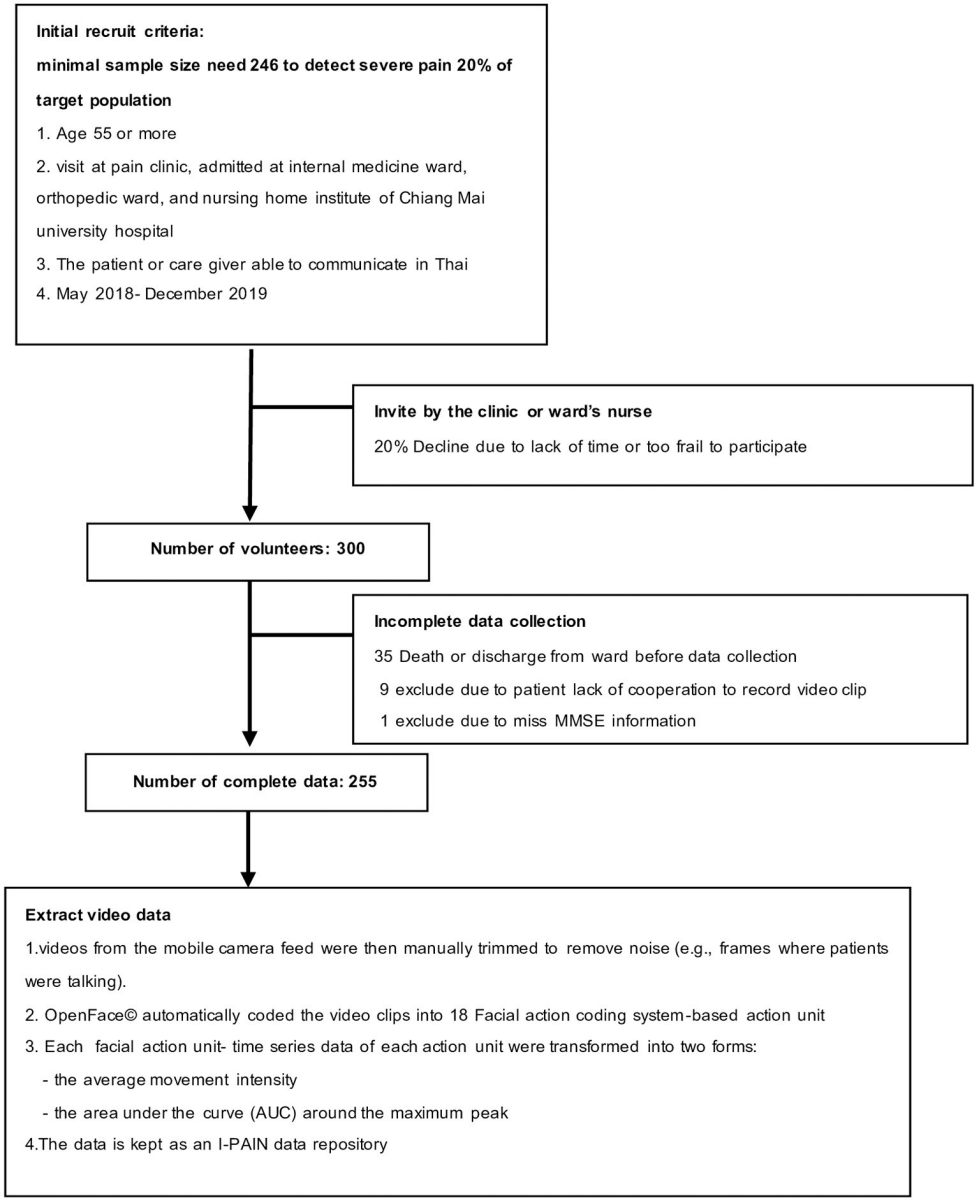
The study flow diagram.

Research assistants recruited participants on weekdays between 9 AM and 4 PM. The data collection questionnaire consisted of demographic questions, and the cognitive status of each patient was evaluated using the minimal mental status (MMSE) Thai 2002 (Boonkerd et al., 2003). The facial expression data were recorded using a Samsung S9 phone's camera for 10 s at a one-meter distance. Patients who could communicate were asked to report their level of pain just before the video clip was recorded. The pain information includes the location, quality, and self-rating severity of the pain using the visual analog scale and Wong–Baker face scale. We recorded the video of non-communicable patients during bed bathing, moving, or having their blood pressure taken, to observe whether these procedures illicit pain behavior. A research assistant trained in pain assessment in dementia (PAINTED) was assigned to rate the video clips of both patients who can and cannot communicate. The PAINAD is a simple score based on five observational domains of pain behavior, including breathing, negative vocalization, facial expression, body language, and consolation (Warden et al., 2003). A total of 255 samples were finally used for the data analysis after 35 could not participate owing to death or discharge from the ward before data collection, and nine were excluded because the patient did not give consent to participate in the study. Details of the study flow are shown in Figure 1.

### Importing and preparing the dataset

Next, background noise, such as frames where patients were talking, was manually removed from the videos from the mobile camera feed. OpenFace^©^ was used to automatically code the video clips into 18 FACS-based AUs. The data are kept as a data repository for further research.

Pain severity was identified as the target class variable of this study using a self-rating WBS. The ratings were as follows: mild between zero and two, moderate between four and six, and severe between eight and ten. A trained research assistant used a PAINAD rating to categorize the pain level of patients who could not communicate. A one-hot encoding (Ramasubramanian and Moolayil, 2019) was used to perform pre-modeling. The ratings were as follows: mild between zero and one, moderate between two and four, and severe above five (Gomutbutra et al., 2020). The FAU-time series data of each action unit, which represented each patient's facial movement over time, were generated using the OpenFace^©^ and were transformed into two forms: (1) the average movement intensity (Sikka, 2014) and (2) the area under the curve (AUC) surrounding the maximum peak (Haines et al., 2019). The AUC of each action unit was calculated using the data from 22 frames (0.03 s per frame) around the maximum peak. These two datasets were then examined to see whether the AUs were related to the level of pain and whether they could be used as characteristics to classify the pain intensity.

Demographic data, such as age, gender, and dementia, were identified as missing values. One case was deleted owing to a lack of MMSE information. Age was categorized into four groups: <60, 61–70, 71–80, and over 80. Gender was coded as 0 for females and 1 for males. Dementia was classified using the MMSE cut point, with a score of 18 or lower for those who only completed a lower education level and a score of 22 for those who completed a higher education level.

### Descriptive analysis

The statistical analysis and the production of figures were performed using R studio version 1.3 (R Core Team, 2014) and MATLAB version 7.0 (MATLAB, 2010), respectively. Demographic data were summarized as percentages, means, medians, and standard deviations. Each AU grouping was compared with the group of facial anatomical movements using correlation analysis with a correlation of 0.3. The correlation of each FAU to pain severity was explored using one-way ANOVA with a defined statistical *p*-value <0.05.

### Classification models and model evaluation

The WEKA software (Frank et al., 2016) was used for data mining. A total of 255 samples were split into training and test data sets in the ratio of 70: 30 (180: 75). The unbalanced data were sampled using the synthetic minority oversampling technique (SMOTE). 10-fold cross-validation was used to select attributes. The models were built using five commonly used classification machine learning techniques, including the generalized linear model, the multilayer perception, which is a subtype of the artificial neural network, J48 decision tree, naïve Bayes, k-nearest neighbors (KNN)—with an optimized K number of 10 in this study—and a sequential minimal optimization support vector machine (SVM). During the model evaluation, ten iterations of 10-fold cross-validation were used in each data set, and the models' overall classification accuracy percentages were compared.

## Results

### Descriptive analysis

A total of 255 Thai communicable elder participants were assessed. The mean age was 67.72 years (SD 10.93, range 60–93). More than half (55%) of patients were male. The majority (90%) had completed more than 4 years of formal education. Nearly all (98%) practiced Buddhism. Approximately 10% had bed-bound functional status. Approximately 47 participants were diagnosed with cancer. Of the 255 elderly participants, 23% met the dementia diagnostic criteria (MMSE of <18). The patients were classified into three categories: moderate pain (55.4%), severe pain (24.4%), and mild pain (20.2%). Back pain was the most frequently experienced (33.8%). Lancinating or “sharpshooting” pain was the most prevalent type (40.2%). The patients' demographic details are shown in Table 1.

**Table 1:** Demographic data (*N* = 255).

**Characteristic**	***N* (SD or %)**
Age [mean (SD)]	67.72 (10.39)
Sex = Male (%)	140 (54.9)
**Education (%)**
No or < 4 years	27 (10.8)
Less than a college degree	112 (45.0)
College degree or above	110 (44.2)
**Religious (%)**
Buddhism	250 (98.0)
Christian	2 (0.8)
Islam	3 (1.2)
**Physical status (%)**
Walking	162 (63.5)
Wheelchair	66 (25.9)
Bed bounded	27 (10.6)
**Underlying disease**
Cancer (%)	114 (47.1)
Diabetes (%)	54 (21.5)
Depression (%)	31 (12.4)
Dementia (%)	60 (23.5)
**MMSE score (%)**
0–10: severe dementia	17 (6.9)
11–18: moderate dementia	37(15.0)
19–25: mild dementia	101 (41.1)
26–30: no dementia	91 (37.0)
**Pain severity (%)**
Mild	52 (20.2)
Moderate	141 (55.4)
Severe	57 (22.4)
**Site of pain**
Head (%)	16 (4.1)
Face (%)	14 (3.6)
Chest (%)	30 (14.9)
Abdomen (%)	44 (17.4)
Back (%)	80 (33.8)
Hip (%)	11 (5.1)
Knee (%)	31 (11.8)
Foot (%)	24 (9.8)
**Quality of pain**
Burning (%)	63 (23.8)
Troubling (%)	73 (28.4)
Lancinating (%)	112 (41.2)
Dull (%)	18 (8.2)
Sore (%)	40 (14.5)
Spastic (%)	52 (20.7)
Paresthesia/difficult to explain (%)	19 (7.7)

**Figure 2:**
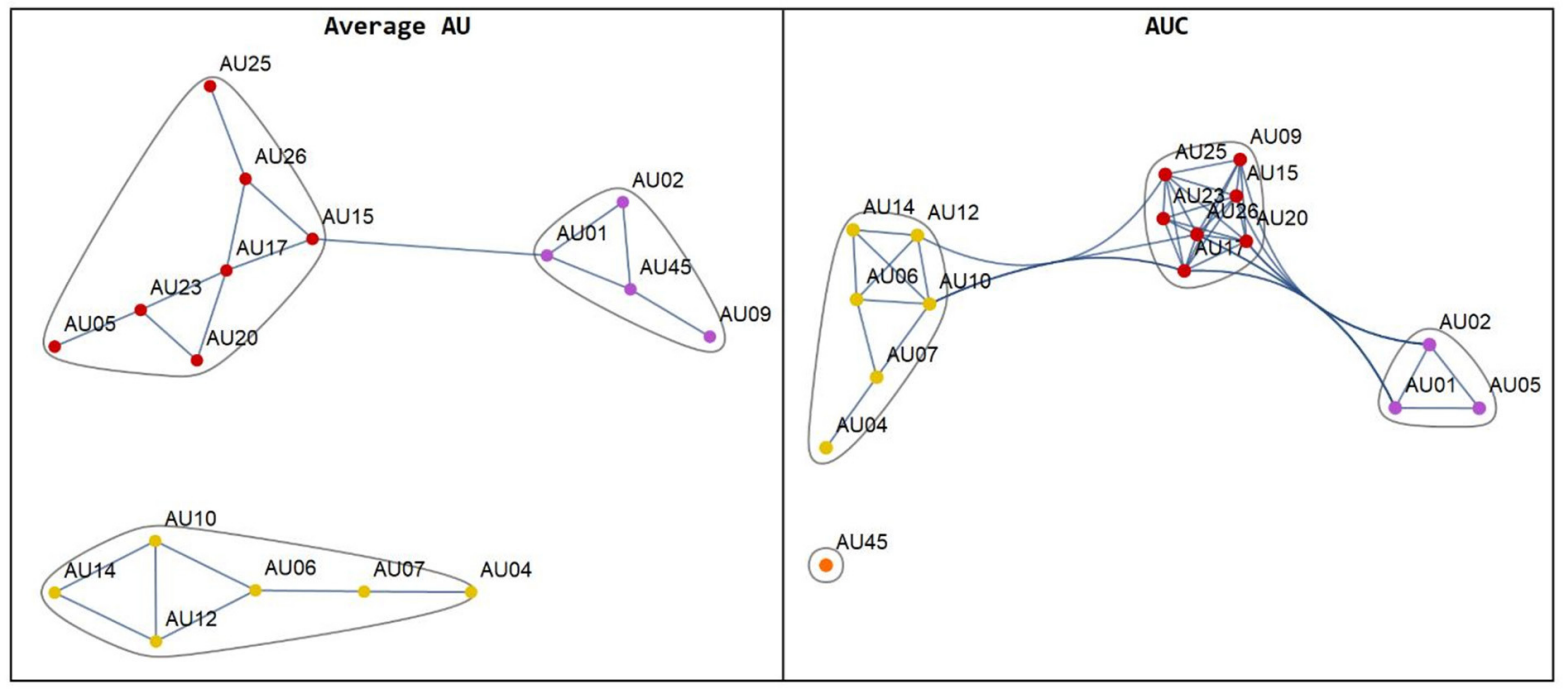
The network plot of the AUs from the average AU (left hand) and the area under the curve: AUC (right hand).

Pearson's correlation coefficient was used to determine the correlation between different AUs. A weighted adjacency graph was created for highly correlated AUs with Pearson's coefficients <0.3, as shown in Figure 2. In the Supplementary Data 1, 2 tables, association information is displayed.

We used ANOVA to analyze the difference between the means of FAUs across the three pain intensity groups. Significant differences were noted in the average activities of AU4 (*p* = 0.04), AU7 (*p* = 0.005), AU10 (*p* = 0.03), and AU25 (*p* = 0.005) from the results of the AUC approach, which identified AU23 (*p* = 0.0045). The Supplementary Data 3–5 were the box plots that represented these comparisons.

### Classification models and model evaluation

Building pain severity classification models use two sources of features: pain-related AUs that have been consistently identified in previous studies (AUs 4, 6, 7, 9, 10, 25, 26, 27, and 45) and selection by machine learning. Features selected using each machine learning method are shown in Supplementary Data 6 and Table 2 shows the accuracy of each machine learning model. Machine-selected features provide the best accuracy. The SVM model for average activities of AU1, 2, 4, 7, 9, 10, 12, 20, 25, and 45, and gender had an accuracy of 58%. The KNN model, which had an accuracy of 56.41% and measures the AUC of AU1, AU2, AU6, AU20, and female, is the second most accurate model. Multilayer perception (50%) and KNN (44.87%) have the highest level of accuracy among the features selected from pain-related FAU in earlier studies.

**Table 2:** The features selected by each machine learning method.

**Classification**	**Selected features**	
	**Average**	**AUC**
Logistic regression	Gender, Dementia, AU01, AU02 AU06, AU09, 10 AU10, AU12 AU20, AU23, AU25	Dementia, AU01,AU02, AU04, AU06 AU07, AU09, AU10, AU12 ,AU14 AU17, AU20
Multilayer perceptron	Gender, Dementia, AU01, AU04, AU05 AU06, AU07, AU09, AU10, AU12 AU14, AU15, AU17, AU20, AU23 AU25,AU26, AU45	Gender, AU05, AU07, AU10,AU12 AU14, AU23, AU25, AU26, AU45
Decision tree	AU01, AU04, AU05, AU06, AU07, AU12, AU14	Gender, AU4, AU12, AU15, AU45
Naïve Bayes	Gender, AU9, AU12	Gender, AU4,AU12,AU15, AU45
KNN (*K* = 10)	Gender, AU7, AU9, AU20, AU25	Gender, AU1, AU2, AU6, AU20
SVM	Gender, AU01, AU02, AU04, AU07 AU09, AU10, AU12, AU20, AU25, AU45	Gender, AU20, AU45

According to the confusion matrix between pain classified as mild, moderate, and severe by either WBS or PAINAD (actual severity) using the SVM and average value from the OpenFace^©^ model, moderate pain is misclassified more frequently than mild or severe pain. The ROC areas for mild, moderate, and severe pain are 0.514, 0.408, and 0.496, and the corresponding F statistics are 0.651, 0.333, and 0.560, as shown in Table 3. The model accuracy for correlation between algorithm-determined PSPI score and self-rating pain severity (no pain, mild, moderate, and severe) is categorized by age groups in Supplementary Data 7. It shows non-significant correlation (*n* = 250, *r* = 0.12; *p* = 0.39). The classification performance is nearly similar for all age groups.

**Table 3:** Confusional matrix between actual severity and model classification for the task classify pain severity.

**Actual severity**	**Model classify**				
	**Mild**	**Moderate**	**Severe**	**ROC**	**F measure**
				**area**	
Mild	85	3	10	0.496	0.651
Moderate	46	23	30	0.408	0.333
Severe	13	32	54	0.514	0.560

## Discussion

The model developed from OpenFace shows no robust classification of pain severity (mild, moderate, and severe) for chronic pain in Asian elders. The best model is SVM for average activities of AU1, 2, 4, 7, 9, 10, 12, 20, 25, and 45, and gender, which had the best accuracy, at 58%. This result was expected and is consistent with previous studies (Lautenbacher et al., 2022). However, this real-world study provided insight into the interpretation and expression issues that continue to pose challenges for automated facial pain ratings. First, the ground truth questions that are reliable on the frame-to-frame facial action unit movement. Second, the cultural influences and sets on the facial pain expression.

### Classification model

Most of the participants in our study were Thai elderly people who were visiting a tertiary care hospital because of chronic pain issues. One-fifth met the MMSE's dementia diagnostic criteria. In the network graph, the closely related AUs, called co-existent, are grouped. The AUC approach provided a more accurate grouping than the average approach. For example, AU1, AU2, and AU5 were anatomically related and were acknowledged as co-existing in a previous study (Peng and Wang, 2018). Because a dependent relationship between each FAU was discovered, regression may not be appropriate for predicting pain severity. However, there is a relationship between the average activity of AU4, AU7, AU10, and AU25 and AUCs of AU17, 23, and dementia, gender, and pain severity. Therefore, these features were included in the classification model. Pain severity-related FAUs were defined in two ways. The systematic review (Kunz et al., 2019) and machine learning selection of the AUs “consistently” described pain-related features. We consistently overlapped AUs from these two methods, such as AU4, AU7, AU10, and AU45, which have already been described in human coding studies (Prkachin and Solomon, 2008).

In addition, machine learning showed the contribution of gender and dementia but did not make the model applicable to older patients. This is consistent with earlier studies that suggested that gender influences how intensely people express their pain when they are more expressive (Taati et al., 2019) and possibly have fairer skin, which influences model accuracy in facial landmark detection (Buolamwini and Gebru, 2018). Previous studies have shown that elders with dementia tend to exhibit more activity around their mouths than in their upper faces (Lautenbacher and Kunz, 2017). According to the confusion matrix, high misclassification in moderate pain is more accurate than obvious mild or severe pain. This nature of the pain classification model was previously discussed in a UNBC McMaster study on the accuracy of OpenFace^©^ to classify pain severity (Sikka, 2014). Therefore, it might not be feasible to use the current automated pain severity classification model for critical decision-making, such as adjusting the opioid analgesics dosage. However, it might be preferable to augment grossly triage tasks, such as supporting evidence of self-rating severity.

### Strength, limitation, and the further implication

This study is one of the few in-depth facial recognition studies on the elderly Asian population. In addition, this study was conducted in a natural setting where stakeholders benefit from the solution. The information from our research may fill the current model's representative bias. Furthermore, it deals directly with the issue of the need for a reasonable increase in accuracy in the current open-source facial analysis software and classification models to classify pain in the elderly. We also produced academically accessible data reciprocity to enhance further model optimization and validation.

This study provides insight into the obstacles to automated facial pain research and possible solutions to overcome them. The caveat of interpretation concerned whether human judgment ratings could be replaced by the value generated by an automated facial recognition algorithm. We estimated the value using the time-event series produced by OpenFace^©^. The algorithm was developed using the UNBC McMaster dataset, which has 80% frames devoid of any indication of pain (Lucey et al., 2011). Given this, OpenFace^©^ might be effective at distinguishing between pain and no pain, but its accuracy in classification pain intensity is debatable. However, merely distinguishing between pain and no pain is insufficient for clinical decision-making when using automated pain assessment in healthcare. We compared two value transform methods: the average method, which theoretically could be influenced by “no pain” frames, and the AUC approach, where the activity correlates strongly with facial muscle anatomical movement and may mitigate this effect. However, our study shows no benefit in using this approach. Although the pain-related FAUs are well described, there is still no consensus on whether the pain severity could depend on the frame-to-frame facial action unit movement (Prkachin and Hammal, 2021). To address this important defect, further research is required to explore the value-generated association between computer vision and rating by a trained rater.

Limitations in generalizability could also prevent the algorithm from being used in clinical settings. The reliability of automated pain severity classification is still not robust enough for medication dosage decision for every ethnic group. Few studies have explicitly trained and tested classifiers on various population databases (Prkachin and Hammal, 2021). Our study discovered many misclassifications of moderate to severe pain into “no pain.” This may explain the spontaneous pain nature, whose behavior expression is significantly influenced by culture and environment. Although a study demonstrated similar facial expressions during pain in Westerners and Asians (Chen et al., 2018), this finding might not indicate a similar degree of expression in particular pain intensity. According to some empirical evidence regarding the social context of pain expression, being around people or interacting with them can affect how much pain is expressed (Krahé et al., 2013). Currently, available data reciprocity including ours was insufficient to address the social context issue. Therefore, there is a greater need for algorithm training using datasets from various countries. An alternative approach involving “individualized” pain behavior pattern recognition may be more practical than using population data to estimate pain severity.

The advance in deep learning methods such as long short-term memory (LSTM) recurrent neural networks seems promising to detect temporal muscle activity (Ghislieri et al., 2021). Anyway, every approach algorithm will require extensive retraining, cross-validating, and the addition of social factors that may improve the model's accuracy and feasibility. International collaboration in transferred learning and fine-tuning algorithm, as well as accessible and sharable data reciprocity, will help accelerate the clinical usability of automated facial recognition.

## Conclusion

Our study on open-source automatic video clip's FAUs' analysis in Thai elders who visited a university hospital is not robust in classifying elder pain. This finding may provide evidence for the need for algorithm training using datasets from various countries. Retraining FAU algorithms, enhancing frame selection strategies, and adding pain-related functions may improve the model's accuracy and feasibility. International collaboration to support accessible and sharable data reciprocity is required to enhance this field.

## Data availability statement

The datasets presented in this study can be found in online repositories. The names of the repository/repositories and accession number(s) can be found below: The CSV file can be downloaded at https://w2.med.cmu.ac.th/agingcare/indexen.html.

## Ethics statement

The study was approved by the Faculty of Medicine Chiang Mai University—CMU IRB No 05429 Institutional Review Board and a waiver for informed consent was approved, allowing for retrospective data anonymization. The patients/participants provided their written informed consent to participate in this study. Written informed consent was obtained from the individual(s) for the publication of any potentially identifiable images or data included in this article.

## Author contributions

PG: main contributor to all aspects of the manuscript. NC and AS: significant contributor to the artificial intelligent methods. SS: create tables, figures by using statistical software. PF: significant contributor to patients recruitment and administration. AK, PS, and PL: are main supervisor in the clinical aspects of the manuscript and contributions to the introduction and discussion. All authors contributed to the article and approved the submitted version.

## Funding

This work was supported by a grant received from the Thai Society of Neurology 2019 and a publication fee granted by Chiang Mai University.

## Conflict of interest

The authors declare that the research was conducted in the absence of any commercial or financial relationships that could be construed as a potential conflict of interest.

## Publisher's note

All claims expressed in this article are solely those of the authors and do not necessarily represent those of their affiliated organizations, or those of the publisher, the editors and the reviewers. Any product that may be evaluated in this article, or claim that may be made by its manufacturer, is not guaranteed or endorsed by the publisher.
