# Supplementary material for: Classification of elderly pain severity from automated video clip facial action unit analysis: A study from a Thai data repository
Source: Front Artif Intell. 2022 Oct 6;5:942248. doi: 10.3389/frai.2022.942248 (PMC9582446; doi:10.3389/frai.2022.942248)
Supplement: Supplementary file 1 [file Data_Sheet_1.PDF]

	AU01	AU02	AU04	AU05	AU06	AU07	AU09	AU10	AU12	AU14	AU15	AU17	AU20	AU23	AU25	AU26	AU45
AU01	1.	0.613	0.0126	-0.0371	-0.00359	0.0586	-0.0111	0.0162	-0.0321	-0.0282	0.255	0.207	0.202	0.0663	0.125	0.143	0.35
AU02	0.613	1.	-0.0937	0.0408	-0.0601	-0.0197	0.0663	-0.0467	-0.000594	-0.0601	-0.0169	-0.0178	0.058	0.179	0.134	-0.0287	0.381
AU04	0.0126	-0.0937	1.	-0.0678	0.218	0.287	0.0389	0.239	-0.00383	0.15	0.00342	-0.0341	-0.0341	-0.02	0.211	0.0813	-0.0355
AU05	-0.0371	0.0408	-0.0678	1.	0.014	-0.118	-0.0255	0.0132	0.0909	-0.0174	-0.0486	-0.0464	-0.0692	0.303	-0.0284	0.0216	-0.19
AU06	-0.00359	-0.0601	0.218	0.014	1.	0.422	0.0791	0.447	0.544	0.234	0.0153	0.158	0.124	0.215	0.14	0.109	-0.0579
AU07	0.0586	-0.0197	0.287	-0.118	0.422	1.	0.014	0.208	0.0543	-0.0376	-0.084	-0.0609	0.146	0.0447	0.132	-0.00195	0.103
AU09	-0.0111	0.0663	0.0389	-0.0255	0.0791	0.014	1.	0.0901	0.0584	-0.000892	0.0912	0.128	0.0895	0.157	0.197	0.0259	0.293
AU10	0.0162	-0.0467	0.239	0.0132	0.447	0.208	0.0901	1.	0.398	0.281	0.109	0.151	0.0172	0.109	0.0779	0.175	-0.0109
AU12	-0.0321	-0.000594	-0.00383	0.0909	0.544	0.0543	0.0584	0.398	1.	0.425	0.026	0.09	-0.00141	0.206	0.0516	0.0818	-0.0523
AU14	-0.0282	-0.0601	0.15	-0.0174	0.234	-0.0376	-0.000892	0.281	0.425	1.	0.0931	-0.00182	-0.0288	0.0409	0.0395	0.2	-0.0421
AU15	0.255	-0.0169	0.00342	-0.0486	0.0153	-0.084	0.0912	0.109	0.026	0.0931	1.	0.4	0.201	0.0617	0.0736	0.322	0.0315
AU17	0.207	-0.0178	-0.0341	-0.0464	0.158	-0.0609	0.128	0.151	0.09	-0.00182	0.4	1.	0.563	0.441	-0.0121	0.266	-0.0521
AU20	0.202	0.058	-0.0341	-0.0692	0.124	0.146	0.0895	0.0172	-0.00141	-0.0288	0.201	0.563	1.	0.347	0.102	0.102	0.0967
AU23	0.0663	0.179	-0.02	0.303	0.215	0.0447	0.157	0.109	0.206	0.0409	0.0617	0.441	0.347	1.	0.0598	0.18	-0.0455
AU25	0.125	0.134	0.211	-0.0284	0.14	0.132	0.197	0.0779	0.0516	0.0395	0.0736	-0.0121	0.102	0.0598	1.	0.31	0.162
AU26	0.143	-0.0287	0.0813	0.0216	0.109	-0.00195	0.0259	0.175	0.0818	0.2	0.322	0.266	0.102	0.18	0.31	1.	0.0372
AU45	0.35	0.381	-0.0355	-0.19	-0.0579	0.103	0.293	-0.0109	-0.0523	-0.0421	0.0315	-0.0521	0.0967	-0.0455	0.162	0.0372	1.
