# Supplementary material for: Classification of elderly pain severity from automated video clip facial action unit analysis: A study from a Thai data repository
Source: Front Artif Intell. 2022 Oct 6;5:942248. doi: 10.3389/frai.2022.942248 (PMC9582446; doi:10.3389/frai.2022.942248)
Supplement: Supplementary file 2 [file Data_Sheet_2.PDF]

	AU01	AU02	AU04	AU05	AU06	AU07	AU09	AU10	AU12	AU14	AU15	AU17	AU20	AU23	AU25	AU26	AU45
AU01	1.	0.736	0.13	0.361	0.084	0.134	0.191	0.13	0.125	0.161	0.27	0.349	0.373	0.233	0.217	0.263	0.204
AU02	0.736	1.	0.0875	0.354	0.0454	0.078	0.291	0.145	0.144	0.215	0.23	0.312	0.403	0.21	0.237	0.299	0.0769
AU04	0.13	0.0875	1.	0.138	0.221	0.272	0.0959	0.21	0.0124	0.143	0.161	0.186	0.0916	0.115	0.197	0.127	-0.0311
AU05	0.361	0.354	0.138	1.	0.102	0.151	0.206	0.137	0.165	0.117	0.159	0.217	0.218	0.238	0.25	0.245	0.0899
AU06	0.084	0.0454	0.221	0.102	1.	0.463	0.165	0.473	0.534	0.289	0.13	0.177	0.147	-0.022	0.182	0.0761	0.0604
AU07	0.134	0.078	0.272	0.151	0.463	1.	0.0865	0.312	0.163	0.0125	0.0915	0.15	0.161	0.0958	0.154	0.138	0.134
AU09	0.191	0.291	0.0959	0.206	0.165	0.0865	1.	0.204	0.227	0.231	0.37	0.344	0.364	0.231	0.333	0.298	0.161
AU10	0.13	0.145	0.21	0.137	0.473	0.312	0.204	1.	0.423	0.33	0.232	0.332	0.146	0.163	0.323	0.26	0.107
AU12	0.125	0.144	0.0124	0.165	0.534	0.163	0.227	0.423	1.	0.475	0.185	0.264	0.24	0.0502	0.2	0.131	0.153
AU14	0.161	0.215	0.143	0.117	0.289	0.0125	0.231	0.33	0.475	1.	0.171	0.191	0.164	0.0772	0.107	0.0922	0.0985
AU15	0.27	0.23	0.161	0.159	0.13	0.0915	0.37	0.232	0.185	0.171	1.	0.439	0.458	0.42	0.421	0.388	0.0696
AU17	0.349	0.312	0.186	0.217	0.177	0.15	0.344	0.332	0.264	0.191	0.439	1.	0.392	0.391	0.473	0.533	0.137
AU20	0.373	0.403	0.0916	0.218	0.147	0.161	0.364	0.146	0.24	0.164	0.458	0.392	1.	0.312	0.429	0.411	0.051
AU23	0.233	0.21	0.115	0.238	-0.022	0.0958	0.231	0.163	0.0502	0.0772	0.42	0.391	0.312	1.	0.408	0.497	0.0768
AU25	0.217	0.237	0.197	0.25	0.182	0.154	0.333	0.323	0.2	0.107	0.421	0.473	0.429	0.408	1.	0.577	0.0781
AU26	0.263	0.299	0.127	0.245	0.0761	0.138	0.298	0.26	0.131	0.0922	0.388	0.533	0.411	0.497	0.577	1.	0.0878
AU45	0.204	0.0769	-0.0311	0.0899	0.0604	0.134	0.161	0.107	0.153	0.0985	0.0696	0.137	0.051	0.0768	0.0781	0.0878	1.
