# Supplementary material for: Classification of elderly pain severity from automated video clip facial action unit analysis: A study from a Thai data repository
Source: Front Artif Intell. 2022 Oct 6;5:942248. doi: 10.3389/frai.2022.942248 (PMC9582446; doi:10.3389/frai.2022.942248)
Supplement: Supplementary file 3 [file Data_Sheet_3.PDF]

Supplementary 3.The means of the action unit values from all patients at different pain levels.

AU	Average AU			AUC		
	Mild	moderate	severe	mild	moderate	severe
AU01	0.12	0.2	0.16	0.51	0.55	0.74
AU02	0.08	0.08	0.06	0.3	0.34	0.45
AU04	1.01	1.05	1.23	1.6	1.79	1.8
AU05	0.06	0.04	0.04	0.17	0.19	0.21
AU06	0.61	0.54	0.61	1.01	0.96	0.91
AU07	1.31	1.25	1.73	2.05	2.24	2.56
AU09	0.04	0.05	0.08	0.21	0.23	0.23
AU10	0.98ab	0.78b	1.05a	1.62	1.48	1.67
AU12	0.43	0.4	0.4	0.72	0.71	0.68
AU14	0.75	0.66	0.7	1.33	1.2	1.32
AU15	0.11	0.18	0.15	0.59	0.47	0.68
AU17	0.39	0.39	0.33	0.81ab	0.76b	0.99a
AU20	0.08	0.12	0.15	0.36	0.41	0.47
AU23	0.14	0.09	0.15	0.3b	0.39ab	0.59a
AU25	0.18a	0.22a	0.43b	0.73	0.75	0.94
AU26	0.32	0.31	0.36	0.88	0.95	1.04
AU45	0.15	0.15	0.23	0.56	0.66	0.68

Data were analyzed by ANOVA; means in columns followed by the same letter are not significantly different according to Bonferroni's test at $\alpha = 0.05$.
