# Supplementary figures and images for: Classification of elderly pain severity from automated video clip facial action unit analysis: A study from a Thai data repository
Source: Front Artif Intell. 2022 Oct 6;5:942248. doi: 10.3389/frai.2022.942248 (PMC9582446; doi:10.3389/frai.2022.942248)

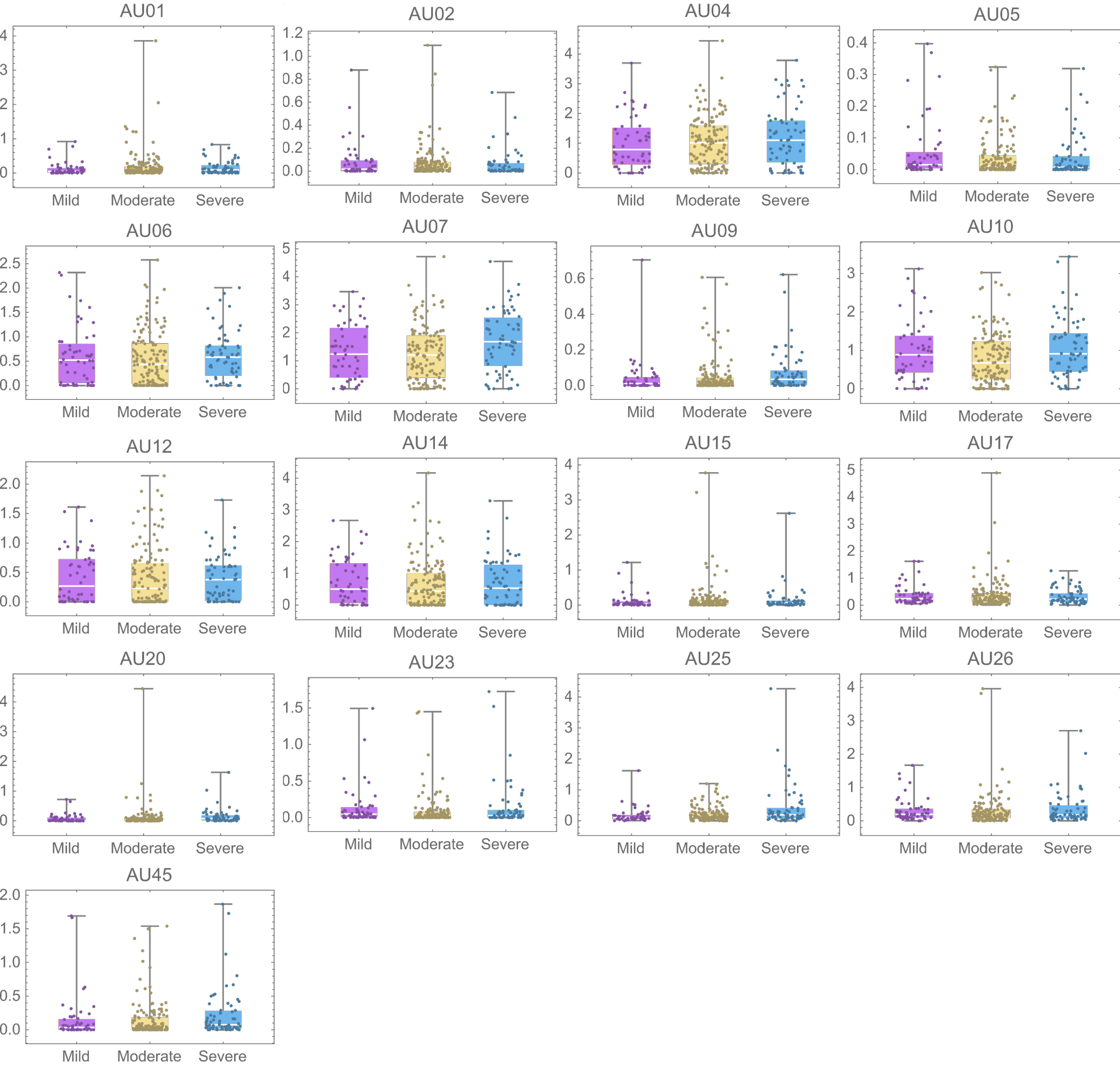

Supplement: Supplementary file 4 [file Data_Sheet_4.PDF]

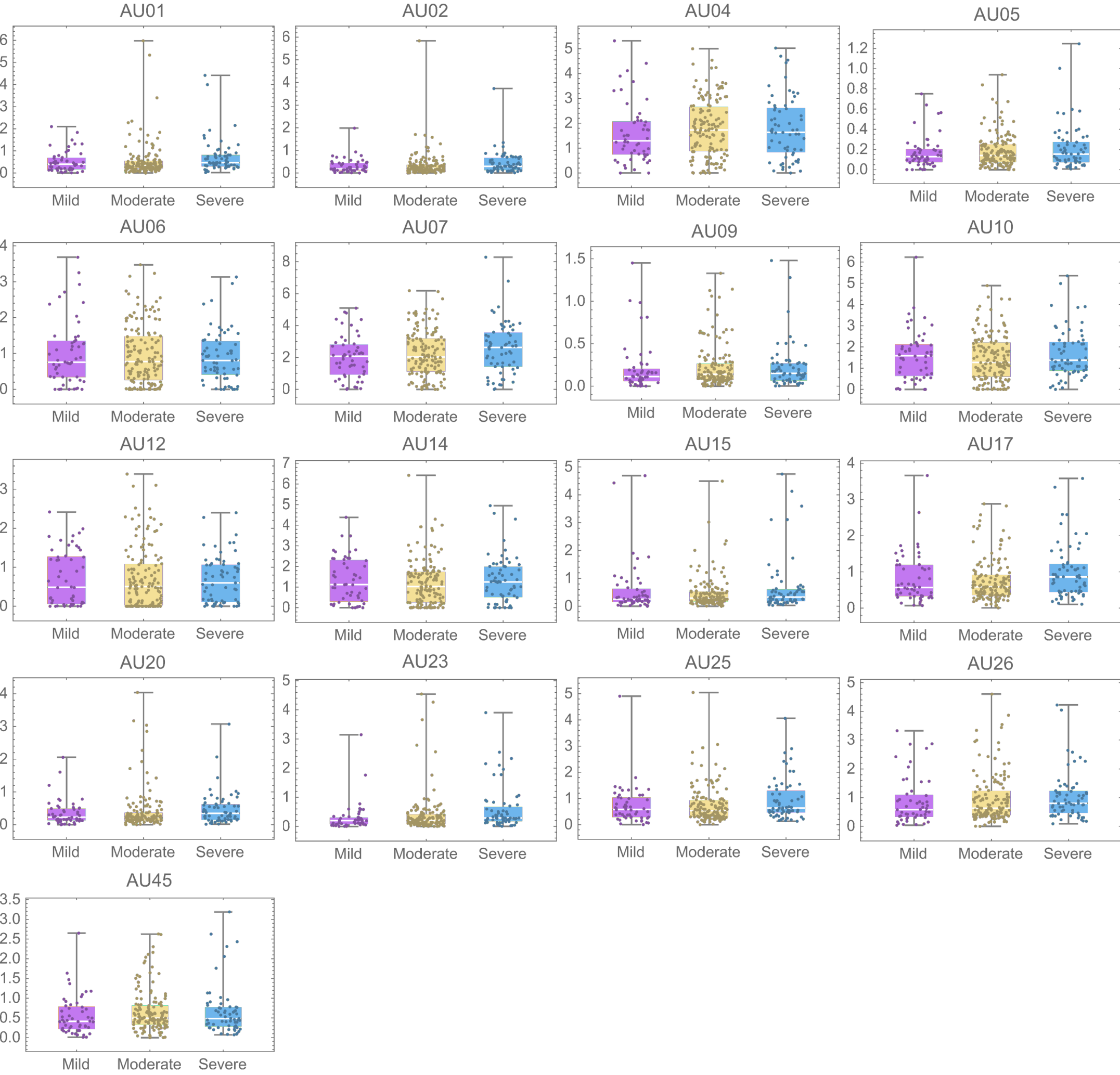

Supplement: Supplementary file 5 [file Data_Sheet_5.PDF]

Supplementary 7 :

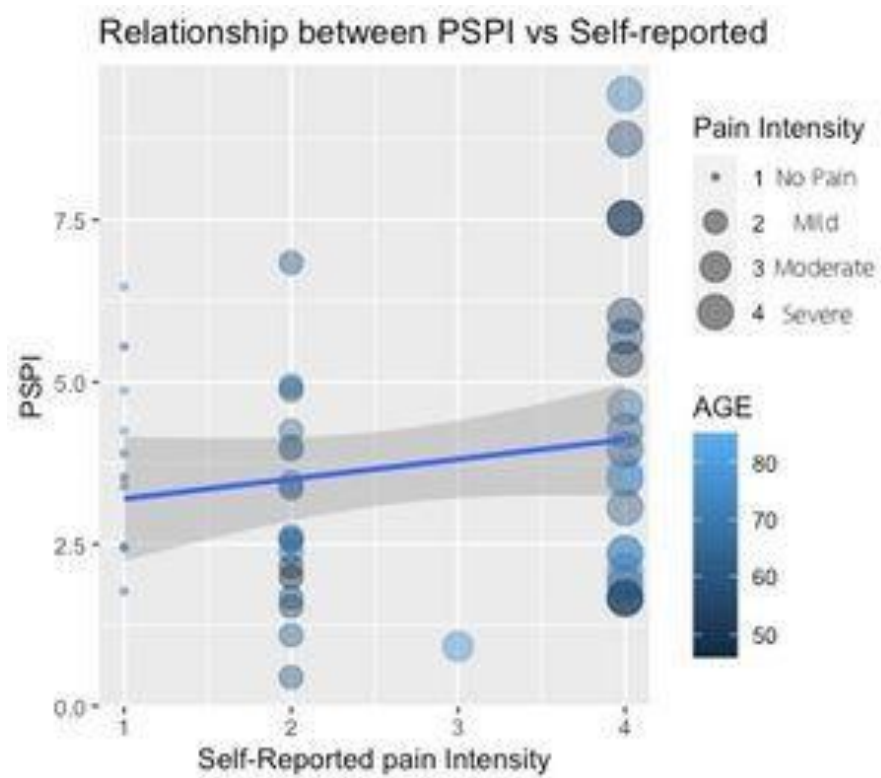

Supplement: Supplementary file 6 [file Data_Sheet_6.PDF]
